# Genetic Susceptibility to Cardiac and Digestive Clinical Forms of Chronic Chagas Disease: Involvement of the *CCR5* 59029 A/G Polymorphism

**DOI:** 10.1371/journal.pone.0141847

**Published:** 2015-11-23

**Authors:** Amanda Priscila de Oliveira, Cássia Rubia Bernardo, Ana Vitória da Silveira Camargo, Luiz Sérgio Ronchi, Aldenis Albaneze Borim, Cinara Cássia Brandão de Mattos, Eumildo de Campos Júnior, Lílian Castiglioni, João Gomes Netinho, Carlos Eugênio Cavasini, Reinaldo Bulgarelli Bestetti, Luiz Carlos de Mattos

**Affiliations:** 1 Laboratório de Imunogenética, Departamento de Biologia Molecular, Faculdade de Medicina de São José do Rio Preto, Avenida Brigadeiro Faria Lima, 5416, 15090–000, São José do Rio Preto, SP, Brazil; 2 Departamento de Cirurgia, Faculdade de Medicina de São José do Rio Preto, Avenida Brigadeiro Faria Lima, 5416, 15090–000, São José do Rio Preto, SP, Brazil; 3 Departamento de Cirurgia Geral, Fundação Faculdade Regional de Medicina—Hospital de Base, Avenida Brigadeiro Faria Lima, 5544, 15090–000, São José do Rio Preto, SP, Brazil; 4 Departamento de Epidemiologia e Saúde Coletiva, Faculdade de Medicina de São José do Rio Preto, Avenida Brigadeiro Faria Lima, 5416, 15090–000, São José do Rio Preto, SP, Brazil; 5 Centro de Investigação de Microrganismos, Departamento de Doenças Dermatológicas, Infecciosas e Parasitárias, Faculdade de Medicina de São José do Rio Preto, Avenida Brigadeiro Faria Lima, 5416, 15090–000, São José do Rio Preto, SP, Brazil; 6 Departamento de Cardiologia e Cirurgia Cardiovascular, Faculdade de Medicina de São José do Rio Preto, Avenida Brigadeiro Faria Lima, 5416, 15090–000, São José do Rio Preto, SP, Brazil; University of Sao Paulo, BRAZIL

## Abstract

The clinical manifestations of chronic Chagas disease include the cardiac form of the disease and the digestive form. Not all the factors that act in the variable clinical course of this disease are known. This study investigated whether the *CCR5*Δ32 (rs333) and *CCR5* 59029 A/G (promoter region—rs1799987) polymorphisms of the *CCR5* gene are associated with different clinical forms of chronic Chagas disease and with the severity of left ventricular systolic dysfunction in patients with chronic Chagas heart disease (CCHD). The antibodies anti-*T*. *cruzi* were identified by ELISA. PCR and PCR-RFLP were used to identify the *CCR5*Δ32 and *CCR5* 59029 A/G polymorphisms. The chi-square test was used to compare variables between groups. There was a higher frequency of the *AA* genotype in patients with CCHD compared with patients with the digestive form of the disease and the control group. The results also showed a high frequency of the *AG* genotype in patients with the digestive form of the disease compared to the other groups. The results of this study show that the *CCR5*Δ32 polymorphism does not seem to influence the different clinical manifestations of Chagas disease but there is involvement of the *CCR5* 59029 A/G polymorphism in susceptibility to the different forms of chronic Chagas disease. Besides, these polymorphisms do not influence left ventricular systolic dysfunction in patients with CCHD.

## Introduction

Chagas disease, caused by the protozoan *Trypanosoma cruzi*, is endemic in Latin America and, due to migration, it has spread to other countries [1–3]. According to the World Health Organization, it is estimated that about 7 million people are infected with *T*. *cruzi*, especially in Latin America [4–7]. The clinical manifestations of the disease occur approximately two decades after infection with about 30% of infected individuals developing the cardiac form of the disease, chronic Chagas heart disease (CCHD). In this case, chronic heart failure is the most important clinical manifestation of the disease [8]. Dilation of the esophagus (megaesophagus) and/or colon (megacolon), characteristic of the digestive form of the disease, affect about 10% of infected individuals [2,4,6].

The variable clinical course of Chagas disease involves distinct populations of parasites, an inflammatory reaction, and host immune response. Genetic variants of cytokines are involved in the different clinical manifestations of the disease. Chemokines are cytokines formed by small proteins involved in the recruitment of leukocytes to inflammation sites. [9–12]. They act via specific receptors belonging to the superfamily of G protein-coupled receptors with seven transmembrane domains. Several types of chemokine receptors are expressed in leukocytes. The CC chemokine receptor 5 (CCR5), ligand of the CCL3, CCL4, and CCL5 chemokines, is expressed by monocytes, macrophages and T lymphocytes, preferentially on T_H_1 cells [13–16].

Different studies have evaluated the role of CCR5 in Chagas disease. One of them reported an increased CCR5 expression in patients with cardiomyopathy compared to individuals with an indeterminate form of infection [17]. Another study reported that the reduction in the CCR5 expression correlates with the reduction observed in the cardiac function [18]. Polymorphisms in the *CCR5* gene were associated with CCHD [11,19–21]. The aim of the present study was to investigate the *CCR5*Δ32 (rs333) and *CCR5* 59029 A/G (promoter region—rs1799987) polymorphisms of the *CCR5* gene in patients with digestive and cardiac forms of chronic Chagas disease and in uninfected individuals with *T*. *cruzi*. We also evaluated the possible association between the genotypes and alleles with the severity of left ventricular systolic dysfunction (LVSD) in patients with CCHD.

## Material and Methods

### Ethical aspects and patient selection

This study was approved by the Research Ethics Committee of the School of Medicine in São José do Rio Preto (#009/2011). The objectives, laboratory procedures, and all details of the study were explained to the patients and control subjects, and those who agreed to participate signed an informed consent form. Two hundred and forty consecutive male and female patients seen at the Cardiomyopathy Outpatient Service and General Surgery of Hospital de Base of the Fundação Faculdade Regional de Medicina (HB-FUNFARME), São José do Rio Preto, SP, Brazil, were enrolled. The control group consisted of 172 male and female blood donors, with negative serology (hemagglutination and immunofluorescence) for *T*. *cruzi* recruited at the Blood Bank in São José do Rio Preto, SP, Brazil.

### Blood sampling and diagnosis of Chagas disease

A total of 5 mL of peripheral blood was collected by venipuncture from each patient in tubes with ethylenediaminetetraacetic acid (EDTA) and 5 mL of blood in tubes without any anticoagulant. Genomic DNA was extracted from leukocytes. Infection by *T*. *cruzi* was confirmed by enzyme-linked immunosorbent assay (ELISA) according to manufacturer's instructions, performed in duplicate (bioMérieux SA, Brazil).

Seropositive patients underwent clinical evaluation, 12-lead electrocardiogram, 2-dimensional echocardiogram and chest X-rays. Patients were considered to have CCHD when presenting with electrocardiographic or echocardiographic abnormalities consistent with the disease [22]. The echocardiographic abnormality indicative of LVSD was a left ventricular ejection fraction (LVEF) <60% measured by the Teicholz’ method. In cases where LVEF could not be measured by this method, a LVEF < 50% at Radionuclide Ventriculography was used to detect LVSD.

Patients were divided into groups according to LVEF; without LVSD was diagnosed in patients with a LVEF ≥60%, mild LVSD in those with LVEF <60% and ≥40%, and severe LVSD in those with LVEF <40%, with the last group being defined according to the Brazilian guidelines of severe chronic heart disease [23]. In cases where LVEF was measured by Radionuclide Ventriculography, patients with LVEF >50%, LVEF between 30% and 50% and LVEF <30%, indicated normal left ventricular systolic function, mild to moderate, and severe LVSD, respectively.

After the clinical evaluation, patients suspected of having the digestive form of the disease were submitted to anorectal manometry, X-ray of the opaque enema, esophageal manometry and X-ray of the esophagus to confirm the diagnosis. Patients with a mixed form of the disease (cardiac and digestive forms) were excluded.

### Genomic DNA extraction and genotyping of the *CCR5* gene

Genomic DNA was extracted using the PureLinkTM Genomic DNA Mini Kit (Invitrogen, Carlsbad, California, USA) according to the manufacturer's instructions. DNA was quantified and its purity determined by the optical densitometry 260/280 nm ratio in a spectrophotometer (Biotek Instruments™—Epoch®).Polymerase chain reaction (PCR) amplification was performed using the CCR5c (5’-CAA AAA GAA GGT CTT CAT TAC ACC-3’) and CCR5d primers (5’-CCT GTG CCT CTT CTT CTC ATT TCG-3’) to identify the deletion of 32 base pairs of the *CCR5* gene (*CCR5*Δ32). The PCR conditions were performed as described by Huang *et al*. [24].

Each reaction had a final volume of 25 μL containing 3.0 mM MgCl2, 0.2 mM of each dNTP [dATP, dTTP, dCTP, dGTP], 20 pM of each primer, 1 unit of Taq polymerase (Invitrogen) and 2 μL of genomic DNA (100 ng/μL). Amplification conditions were: 40 cycles with the first five cycles at 94°C for 1 minute, 55°C for 1 minute, 72°C for 1.5 minutes, followed by 35 cycles at 94°C for 30 seconds, 61°C for 30 seconds, and 72°C for 45 seconds. The PCR product was observed by gel electrophoresis in 1.2% agarose stained with ethidium bromide and visualized under ultraviolet light. The fragment of 189 base pairs corresponds to the *CCR5* wild allele and that of 157 base pairs corresponds to the deletion allele.

The methodology used to identify the *CCR5* 59029 A/G polymorphism (promoter region of the *CCR5* gene) was Polymerase Chain Reaction and Restriction Fragment Length Polymorphism (PCR-RFLP). The promoter region of the *CCR5* gene was amplified using the sense (5’-CCC GTG AGC CCA TAG TTA AAA CTC-3’) and antisense primers (5’-TCA CAG GGC TTT TCA ACA GTA AGG-3’). The conditions of the PCR-RFLP technique were performed as described by McDermott *et al*. [25].

Each reaction had a final volume of 25 μL containing 2.5 mM MgCl2, 0.2 mM of each dNTP [dATP, dTTP, dCTP, dGTP], 20 pM of each primer, 0.5 unit of Taq polymerase (Invitrogen) and 2 μL of genomic DNA. Each amplification reaction consisted of one cycle at 94°C for 5 minutes, followed by 35 cycles at 95°C for 1 minute, 54°C for 1 minute, 72°C for 1 minute and a cycle of 72°C for 10 minutes. The PCR product containing 268 base pairs was observed by gel electrophoresis in 2% agarose stained with ethidium bromide and visualized under ultraviolet light.

The amplified DNA was digested at 37°C for 30 minutes using the Fast Digest enzyme Bsp1286I (Fermentas-Thermo Scientific). The fragments of 10 base pairs and 258 base pairs correspond to the A allele, while the fragments of 10, 127 and 131 base pairs correspond to the G allele. The fragments were observed by gel electrophoresis in 2% agarose stained with ethidium bromide and visualized under ultraviolet light.

### Statistical analysis

Quantitative variables are expressed as mean, median and standard deviation. The unpaired t-test was used to compare continuous variables. The genotype frequencies were evaluated using the recessive inheritance model (AA vs. AG + GG) and the dominant inheritance model (AA + AG vs. GG). Statistical calculations were performed using GraphPad Instat software (version 3.06). The chi-square test was used to compare proportions between groups, adopting a level of significance of 5%. The Hardy-Weinberg equilibrium was calculated using the BioEstat software (version 5.3).

## Results

Of the 240 patients with positive serology for *T*. *cruzi*, 130 (54.2%) were women and 110 (45.8%) were men. The overall mean age was 64.7 ± 10.9 (median: 65.5; Range: 31–93). One hundred and nine (45.4%) of the patients had the digestive form of Chagas disease and 131 (54.6%) had CCHD. The control group consisted of 172 subjects; 90 (52.3%) were female and 82 (47.7%) were male. The overall mean age was 31.5 ± 11.0 (median: 30.0; range: 18–64). The difference between the mean age of patients with chronic Chagas disease (64.7 ± 10.9) and the control group (31.5 ± 11.0) was statistically significant (p <0.0001).

The genotype frequencies for the *CCR5*Δ32 polymorphism among the 240 patients were 223 (92.9%) with *CCR5*/*CCR5* and 17 (7.1%) *with CCR5*/*CCR5*Δ32. The *CCR5*Δ32/*CCR5*Δ32 genotype was not observed in the group of patients. The control group also showed a high frequency of the *CCR5/CCR5* genotype; one individual was homozygous for the *CCR5*Δ32/*CCR5*Δ32 genotype. Table 1 shows the comparison of genotypes and alleles of the *CCR5*Δ32 polymorphism between patients and controls. The distribution of the genotypes of the *CCR5*Δ32 polymorphism was in Hardy-Weinberg equilibrium in all the three study groups (Digestive Disease: χ2 = 0.060, degrees of freedom [DF] = 1, p = 0.806; Heart Disease: χ2 = 0.302, DF = 1, p = 0.583; Control Group: χ2 = 0.886, DF = 1, p = 0.346).

**Table 1:** Frequency of genotypes and alleles of the *CCR5*Δ32 polymorphism in patients with chronic Chagas disease and control subjects.

Genotypes	Digestive form		Cardiac form		Control		χ^2^	DF^a^	p^b^
*CCR5*Δ32	n	%	n	%	n	%			
*CCR5/CCR5*	104	95.4	119	90.8	156	90.7	2.358	2	0.307
*CCR5/CCR5*Δ32	5	4.6	12	9.2	15	8.7	2.112	2	0.348
*CCR5*Δ32/*CCR5*Δ32	0	0	0	0	1	0.6	1.399	2	0.497
Total	109	100.0	131	100.0	172	100.0			
**Alleles**									
*CCR5*	213	97.7	250	95.4	327	95.1	2.566	2	0.277
*CCR5*Δ32	5	2.3	12	4.6	17	4.9			

^a^ DF: Degrees of freedom

^b^ Calculated by χ^2^.

Genotyping of the *CCR5* 59029 A/G polymorphism was performed in 219 patients with chronic Chagas disease. In all patients, the frequencies of the *AA*, *AG*, and *GG* genotypes were 68 (31.1%), 91 (41.5%) and 60 (27.4%), respectively. In the control group, the genotype frequencies were 47 (28.1%), 74 (44.3%) and 46 (27.5%) for the *AA*, *AG* and *GG*, respectively. The distribution of the *CCR5* 59029 A/G polymorphism genotypes was in Hardy-Weinberg equilibrium in patients with digestive disease (χ2 = 0.001; DF = 1; p = 0.979) and in the control group (χ2 = 2.160; DF = 1; p = 0.142) whereas for the group of patients with CCHD it was outside of the Hardy-Weinberg equilibrium (χ2 = 10.722; DF = 1; p = 0.001). The genotype and allele frequencies of the *CCR5* 59029 A/G polymorphism are shown in Table 2.

**Table 2:** Frequency of genotypes and alleles of the *CCR5* 59029 A/G polymorphism in patients with Chagas disease and control subjects

Genotypes	Digestive form		Cardiac form		Control		χ^2^	DF^a^	p^b^
*CCR5* 59029A/G	n	%	n	%	n	%			
*AA*	22	22.4	46	38.0	47	28.1	6.656	2	0.036
*AG*	49	50.0	42	34.7	74	44.3	5.466	2	0.065
*GG*	27	27.6	33	27.3	46	27.5	0.003	2	0.998
Total	98	100.0	121	100.0	167	100.0			
*AA*	22	22.4	46	38.0			6.129	1	0.013
*AG*	49	50.0	42	34.7			5.212	1	0.022
*GG*	27	27.6	33	27.3			0.002	1	0.963
**Alleles**									
*A*	93	47.4	134	55.4	168	50.3	2.897	2	0.235
*G*	103	52.6	108	44.6	166	49.7			
Genotype comparisons									
Digestive form vs. cardiac form							7.274	2	0.026
Digestive form vs. control group							1.200	2	0.549
Cardiac form vs. control group							3.725	2	0.155

^a^ DF: Degrees of freedom

^b^ Calculated by χ^2^.

A significant difference was found using the recessive inheritance model (*AA* vs. *AG* + *GG*) to compare the genotype frequencies between patients with digestive and cardiac forms of chronic Chagas disease, and the control group (p = 0.036—Table 3).

**Table 3:** *CCR5* 59029 A/G polymorphism analysis using the recessive inheritance model.

Genotypes	Digestive form		Cardiac form		Control		χ^2^	DF^a^	p^b^
*CCR5* 59029A/G	n	%	n	%	n	%			
*AA*	22	22.4	46	38.0	47	28.1	6.656	2	0.036
*AG* + *GG*	76	77.6	75	62.0	120	71.9			
Total	98	100.0	121	100.0	167	100.0			
Genotype comparisons									
Digestive form vs. Cardiac form							6.129	1	0.013
Digestive form vs. control group							1.040	1	0.308
Cardiac form vs. control group							3.128	1	0.077

^a^ DF: Degrees of freedom

^b^ Calculated by χ^2^.

However, no significant difference was observed in the dominant inheritance model (*AA* + *AG* vs. *GG*), between the same groups (p = 0.998—Table 4). Among patients with CCHD, 66 (50.4%) patients had normal left ventricular systolic function, 30 (22.9%) mild to moderate LVSD, and 35 (26.7%) severe LVSD. Table 5 and Table 6 show the comparison of genotypes and alleles of the *CCR5*Δ32 and *CCR5* 59029 A/G polymorphisms between patients with CCHD classified according to the severity of LVSD, respectively.

**Table 4:** *CCR5* 59029 A/G polymorphism analysis using the dominant inheritance model.

Genotypes	Digestive form		Cardiac form		Control		χ^2^	DF^a^	p^b^
*CCR5* 59029A/G	n	%	n	%	n	%			
*AA* + *AG*	71	72.4	88	72.7	121	72.5	0.003	2	0.998
*GG*	27	27.6	33	27.3	46	27.5			
Total	98	100.0	121	100.0	167	100.0			
Genotype comparisons									
Digestive form vs. cardiac form							0.002	1	0.963
Digestive form vs. control group							1.155	1	0.999
Cardiac form vs. control group							0.003	1	0.959

^a^ DF: Degrees of freedom

^b^ Calculated by χ^2^.

**Table 5:** Frequency of genotypes and alleles of the *CCR5*Δ32 polymorphism in patients with chronic Chagas heart disease with normal left ventricular systolic function, mild to moderate, and severe left ventricular systolic dysfunction.

Genotypes	Normal		Mild/Moderate LVSD		Severe LVSD		χ^2^	DF^a^	p^b^
*CCR5*Δ32	n	%	n	%	n	%			
*CCR5/CCR5*	61	92.4	28	93.3	30	85.7	1.528	2	0.466
*CCR5/CCR5*Δ32	5	7.6	2	6.7	5	14.3			
Total	66	100.0	30	100.0	35	100.0			
**Alleles**									
*CCR5*	127	96.2	58	96.7	65	92.9	1.455	2	0.483
*CCR5*Δ32	5	3.8	2	3.3	5	7.1			

LVSD: Left ventricular systolic dysfunction

^a^ DF: Degrees of freedom

^b^ Calculated by χ^2^.

**Table 6:** Frequency of genotypes and alleles of the *CCR5* 59029 A/G polymorphism in patients with chronic Chagas heart disease with normal left ventricular systolic function, mild to moderate, and severe left ventricular systolic dysfunction.

Genotypes	Normal		Mild/Moderate LVSD		Severe LVSD		χ^2^	DF^a^	p^b^
*CCR5* 59029A/G	n	%	n	%	n	%			
*AA*	26	41.3	9	32.1	11	36.7	0.716	2	0.699
*AG*	19	30.1	11	39.3	12	40.0	1.205	2	0.547
*GG*	18	28.6	8	28.6	7	23.3	0.312	2	0.855
Total	63	100.0	28	100.0	30	100.0			
**Alleles**									
*A*	71	56.3	29	51.8	34	56.7	0.381	2	0.827
*G*	55	43.7	27	48.2	26	43.3			

LVSD: Left ventricular systolic dysfunction

^a^ DF: Degrees of freedom

^b^ Calculated by χ^2^.

## Discussion

The aim of this study was to investigate the association of the *CCR5*Δ32 and *CCR5* 59029 A/G polymorphisms in patients with digestive and cardiac forms of chronic Chagas disease. The different clinical courses of Chagas disease are associated with the inflammatory reaction and differences in host immune response [9,11]. Several studies were conducted to investigate the role of CCR5 in the pathogenesis of Chagas disease [17–21]. However, this seems to be the first study evaluating these two *CCR5* polymorphisms in the different clinical forms of chronic Chagas disease.

There was a statistically significant difference in respect to age, with the mean age of the patients in this study being higher than the mean age of the control group. In fact, individuals infected with *T*. *cruzi* develop the chronic forms of Chagas disease about two decades after infection [26], which contributes to the high mean age of patients. Moreover, healthy individuals between 18 and 67 years are two requisites for blood donation in Brazil [27,28], which results in a younger population compared to the patients.

The *CCR5*Δ32 genetic variant results from the deletion of 32 base pairs in the *CCR5* gene, which generates a truncated protein that is missing from the cell surface. In heterozygous individuals, the expression of the CCR5 receptor is reduced [29,30]. The differences in the distribution of genotypes and alleles of the *CCR5*Δ32 polymorphism between patients with CCHD and digestive disease, and the control group were not statistically significant. This lack of association may be due to the low frequency of the *CCR5*Δ32 allele in the population studied. The frequency of this allele is high in European populations, however its frequency in Amerindian populations is rare or absent [31,32]. A study carried out in Brazil also revealed a low frequency of the *CCR5*Δ32 allele and absence of individuals homozygous for this deletion [33,34]. It is worthwhile to mention that other studies found a low prevalence of the *CCR5*Δ32 allele in patients with Chagas disease [19,35].

Although the mobilization of immune cells is essential to reduce the parasitic load, the high production of chemokines as well as the expression of their receptors induces migration of large numbers of inflammatory cells into the myocardium, which act as an effector of heart damage [36–39]. In digestive Chagas disease, there is an association of inflammatory infiltration and fibrosis with lesions found in muscle cells. Immunological differences from the host are involved in the different clinical manifestations of disease [9,37]. The *CCR5*Δ32 polymorphism does not appear to be involved in the differential immune response between patients with digestive and cardiac disease. Therefore, the results of the current study suggest that the *CCR5*Δ32 polymorphism does not influence the differential clinical manifestation of chronic Chagas disease.

The *CCR5*Δ32 polymorphism was analyzed in patients with CCHD [19,35,40,41]. Although the differences were not statistically significant, a study in Venezuela suggested a protective role for the *CCR5*/*CCR5*Δ32 genotype in development of cardiomyopathy [41]. To date, the *CCR5*Δ32 polymorphism has not been evaluated in Chagas digestive disease.

The results of the investigation of the polymorphism of the *CCR5* promoter region (*CCR5* 59029 A/G) identified a higher frequency of the *AA* genotype in patients with CCHD compared with those with chronic Chagas digestive disease and the control group. This difference is evident on comparing the groups using the recessive inheritance model. The results of this study also show a higher frequency of the *AG* genotype in patients with the digestive form of the disease than those of the other groups. This polymorphism affects the CCR5 level of expression on cell surface. Individuals with the *AA* genotype have greater expression of CCR5 on the surface of leukocytes compared with other genotypes, as the *G* allele has less promoter activity in vitro than the *A* allele [25,42].

In CCHD, B and T lymphocytes, macrophages and a few NK cells are the major infiltrating cells, with the CD8^+^ T cells being the predominant cell population; CD4+ TH1 cells are involved in differentiation and activation of CD8^+^ T cells [36,43–45].

The expression of chemokines CCL3, CCL4 and CCL5 was reported during the acute and chronic stages of infection by *T*. *cruzi* [46]. CCL4 preferably attracts the CD4^+^ T cells while CCL3 predominantly attracts CD8^+^ T cells [47]. In mice infected with *T*. *cruzi*, the contribution of the CCL5 to the infiltration of CD8^+^ T cells has been demonstrated [48]. In fact, these CC chemokines preferably attract activated CD8^+^ T lymphocytes resulting in an increased number of these cells in the heart of mice infected with *T*. *cruzi* [49].

A high proportion of CD8^+^ cells expressing CCR5 was observed in mice infected by *T*. *cruzi* [50]. Also, a high expression of CCR5 in CD8^+^ T cells was observed in the peripheral blood of infected patients [18,45]. Together with the expression profile of chemokines, CCR5 may play a role in the predominance of CD8^+^ T cells in the myocardium. Thus, the *AA* genotype may contribute to the intense inflammatory infiltrate and predominance of CD8^+^ T cells in the heart of patients infected by *T*. *cruzi* favoring the development of Chagas myocarditis.

The composition of the inflammatory infiltrate in the digestive forms of Chagas disease includes macrophages, mast cells, eosinophils, CD4^+^ and CD8^+^ T cells, B cell and NK lymphocytes, with the CD4^+^ T cells being the most abundant compared to CD8^+^ T cells [9,51–53]. In humans, a higher percentage of CD8^+^ T cells expressing CCR5 were identified compared to CD4^+^ T cells [54]. The elevated frequency of *AG* genotype in patients with digestive disease observed in this study suggests a reduced inflammatory process dependent of the CCR5 compared to the cardiac form of Chagas disease. Thus, the inflammatory activity in the digestive forms of the disease may be less dependent on the CCR5 receptor and its ligands than that observed in the CCHD.

Denervation is the main pathophysiological mechanism of digestive chronic Chagas disease, which plays an important role in the formation of megaesophagus and megacolon. The cause of the reduction in the number of neurons remains unclear [9]. A high number of mast cells and eosinophils were observed in patients with megacolon and these cells together with macrophages were correlated with the occurrence of fibrosis in the colon of these patients. In this same study, eosinophils were associated with the damaged neuronal ganglia [53]. Mononuclear cells, eosinophils and mast cells were also present in the inflammatory infiltrate of patients with megaesophagus. In addition, periganglionitis and vasculitis were also identified in these patients [52]. Although association of CD8^+^ T cells with degenerated ganglion cells has been reported in patients with megacolon [55] these do not seem to be the predominant cells [51]. Of course, there are other pathophysiological mechanisms and other cells involved in the pathogenesis of digestive Chagas disease.

The *CCR5* 59029 A/G polymorphism was associated with protection in the development of Chagas cardiomyopathy [40,41]. Calzada *et al*. [40] suggested an association of the *G* allele with protection against chagasic cardiomyopathy. The results of another study suggest that the *GG* genotype has a protective role in the development of cardiomyopathy related to *T*. *cruzi* infection [41]. In contrast to the results of this study and the results mentioned above, Flórez *et al*. [19] revealed the presence of the G allele in the haplotype associated with the susceptibility to the chagasic cardiomyopathy. The *CCR5* 59029 A/G polymorphism has not been studied in the digestive form of chronic Chagas disease.

The differences in the distribution of genotypes and alleles of the *CCR5*Δ32 and *CCR5* 59029 A/G polymorphisms between patients with CCHD evaluated according to the degree of LVSD were not statistically significant. The results of this study are consistent with the results reported in another recent study by our group. We investigated these polymorphisms among patients with CCHD with and without LVSD, and found no association between *CCR5*Δ32 and *CCR5* 59029 A/G polymorphisms and left ventricle impairment [35]. Although Talvani *et al*. [18] found that with the presence of cardiac dysfunction, there was a decrease in CCR5 expression, the *CCR5* 59029 A/G polymorphism does not appear to be associated with the severity of CCHD, but with the differential clinical manifestation of the heart and digestive forms.

The expression of CCR5 was evaluated in Chagas disease. Gomes *et al*. [17] showed a lower expression of CCR5 in patients with an indeterminate form of the disease compared with patients with cardiomyopathy. These authors support the hypothesis that the development of heart disease is associated with an increased T_H_1-type immune response in particular. In another study with mice, treatment with Met-RANTES (N-terminal-methionylated RANTES) in the chronic phase of *T*. *cruzi* infection resulted in decreased damage to heart tissue and dysfunction [56]. In addition, a study of cardiac tissue of beagle dogs infected with *T*. *cruzi* suggested that high expression of CCR5 mRNA and myocarditis may be correlated [57].

In this study, for the group of CCHD patients, unlike the control group, the distribution of the genotypes of the *CCR5* 59029 A/G polymorphism is outside of the Hardy-Weinberg equilibrium. Although some authors argue that both groups should be in Hardy-Weinberg equilibrium [58], others argue that this equilibrium should only be investigated in the control group as it represents the general population [59–61]. Therefore, the high frequency of the *AA* genotype might be changing the distribution of this polymorphism in individuals with heart disease, resulting in a deviation from the Hardy-Weinberg equilibrium. Despite the current state of knowledge on the CCR5 receptor especially in cardiac form of chronic Chagas disease, the role of polymorphisms *CCR5*Δ32 and *CCR5* 59029 A/G in different clinical forms of the disease still needs further clarification, especially due to the small number of patients enrolled in this study.

According to our results, the *CCR5*Δ32 polymorphism does not seem to influence the different clinical manifestations of Chagas disease. The results of this study show the involvement of the *CCR5* 59029 A/G polymorphism in susceptibility to chronic forms of Chagas disease in the population studied.

## Conclusions

In conclusion our results show that the *CCR5* 59029 AA genotype is associated with the CCHD and the genotype *AG* is associated with the digestive form of chronic Chagas disease. In addition, these polymorphisms do not influence LVSD in patients with CCHD.
